# Fertility decline and the changing dynamics of wealth, status and inequality

**DOI:** 10.1098/rspb.2015.0287

**Published:** 2015-05-07

**Authors:** Heidi Colleran, Grazyna Jasienska, Ilona Nenko, Andrzej Galbarczyk, Ruth Mace

**Affiliations:** 1 Institute for Advanced Study in Toulouse, Toulouse School of Economics, Toulouse 31015, France; 2 Department of Environmental Health, Jagiellonian University Medical College, Krakow 31-531, Poland; 3 Department of Anthropology, University College London, London WC1H 0BW, UK

**Keywords:** fertility decline, wealth, status, inequality, demographic transition

## Abstract

In the course of demographic transitions (DTs), two large-scale trends become apparent: (i) the broadly positive association between wealth, status and fertility tends to reverse, and (ii) wealth inequalities increase and then temporarily decrease. We argue that these two broad patterns are linked, through a diversification of reproductive strategies that subsequently converge as populations consume more, become less self-sufficient and increasingly depend on education as a route to socio-economic status. We examine these links using data from 22 mid-transition communities in rural Poland. We identify changing relationships between fertility and multiple measures of wealth, status and inequality. Wealth and status generally have opposing effects on fertility, but these associations vary by community. Where farming remains a viable livelihood, reproductive strategies typical of both pre- and post-DT populations coexist. Fertility is lower and less variable in communities with lower wealth inequality, and macro-level patterns in inequality are generally reproduced at the community level. Our results provide a detailed insight into the changing dynamics of wealth, status and inequality that accompany DTs at the community level where peoples' social and economic interactions typically take place. We find no evidence to suggest that women with the most educational capital gain wealth advantages from reducing fertility, nor that higher educational capital delays the onset of childbearing in this population. Rather, these patterns reflect changing reproductive preferences during a period of profound economic and social change, with implications for our understanding of reproductive and socio-economic inequalities in transitioning populations.

## 1. Introduction

The dramatic fertility declines that accompany transitions from subsistence farming to a market economy are typified by two important large-scale patterns: (i) an apparent reversal or dampening of the broadly positive association between wealth, status and fertility [1–3]; and (ii) a short-term reversal in trends in inequality [4–8]. Prior to the demographic transition (DT), wealthy and high status people typically have higher fertility than poorer and lower status people [9–15], though some historical evidence suggests that this relationship is not so straightforward [16,17]. Wealth inequality is moderately high [14,18–21] compared with hunter–gatherers. In transitioning and post-DT populations, by contrast, wealthy and high status people typically have lower fertility than poorer and lower status people [1,3] and wealth inequality is temporarily lower than in pre-DT contexts [4,6,14].

Despite extensive research on how wealth and status influence fertility, there is little agreement about how these two broad reversals occur, or how they may be connected. Many studies focus on either pre-DT [3,9–12,15,22–24] or post-DT populations [1,25–29], where the measures of wealth and status—and the cultural and economic contexts in which they matter—differ dramatically. This heterogeneity makes it difficult to compare the magnitude and variation of effects across study sites [30], or to identify points on a continuum of change [3]. More detailed comparative studies are needed in transitioning populations, where both ‘traditional’ and ‘modern’ forms of wealth and status influence fertility. Changing relationships can then be demonstrated [14,30,31]. However, few studies have compared wealth and status effects on fertility in multiple local contexts [14,32,33], rather than across regions [3,34,35] or countries [3,21,25,27]. Fewer still have examined how interactions between different measures may produce context-dependent reproductive outcomes [27,31,35]. Moreover, little is known about how wealth and status inequality varies on the continuum of economic modernization [3,36–38], or indeed, how inequality relates to fertility in mid-transitional contexts [14,37,39].

At a macro-level, contemporary fertility declines and both income inequality and economic growth are strongly associated with the accumulation of educational capital [4–6,38–40]. Kuznets hypothesized [6] that income inequality increases and then decreases in the course of market integration. This pattern, he argued, emerged purely as a function of underlying structural change—the declining importance of the farming sector and increasing importance of formal schooling—in a shift that increases average income as ever-larger shares of the population embrace market-labour over subsistence farming. Although inequality increases again in late capitalist societies [8,41], macro-level evidence suggests that income inequality generally decreases as fertility declines [5,37] and as average income increases ([5,7], though see [8]). There are little data to support these patterns at lower levels of aggregation, where peoples' social and economic interactions typically take place [14,36]. Yet local contexts should shape reproductive decisions more than should higher-level aggregates [42], because local distributions of resources and opportunities govern what economic activities are possible, and because local social interactions constrain and facilitate acceptable behaviour [43,44]. Increasingly, status competition is thought to occur within rather than between social strata and regions [25,35,45,46]. This makes it important to examine wealth and status effects on fertility relative to local, rather than absolute aggregates. Since macro-level patterns are often used to infer individual decision-mechanisms, we should also learn whether local patterns and inequalities reproduce macro-level ones.

And yet the meanings of wealth and status—not only the effects—are in flux during the course of market integration, as they are in other subsistence transitions [18–21,47,48]. As hunter–gatherers transition to agriculture, the primacy of ‘embodied’ capital in the form of physical size, local knowledge, hunting and fighting skill appears to give way to multiple dimensions of ‘extrasomatic’ capital, such as land, livestock and material assets [20,21,49,50], which positively correlate with fertility [9–13,32]. By contrast, transitioning and post-DT populations are typified by the re-emergence of embodied capital in the form of education, skill accumulation and occupational status as central to socio-economic [38] but not, apparently, reproductive success [49,50]. Definitions of wealth and status vary [9,29,48,49,51], but in evolutionary anthropology, wealth is probably most often understood as ‘resources’ and status as ‘access to resources’ [26,27,29,34,51]. This dichotomy is useful because of its general applicability across contexts (and species) and because it aids in the development of causal hypotheses: we expect status differentials to determine how wealth affects fertility, rather than the other way around.

We argue that as subsistence farmers transition to a market economy, wealth and status become decoupled in their effects on fertility, allowing variation in reproductive strategies to emerge alongside new forms of status stratification. Couples abandoning farming for market-oriented employment receive an economic and a demographic dividend. By increasing labour-force participation, non-farming income is increased, permitting investments in educational capital, savings and other assets, generating marginal advantages in the market economy [38], even for couples who are not educated themselves. And because market-integrated couples may not need (or want) large families, resources are diluted among fewer people. This mirrors a macro-level pattern whereby fertility declines temporarily change the age-structure of a population, allowing for periods of rapid economic growth [41,52]. Farmers who diversify their income sources by having family members in the labour market may still need (or want) more children, so they experience greater dilution of their market-related resources. Small reproductive and income differentials can magnify inequalities between non-farming and farming households. If couples marry assortatively according to education or earning potential, these inequalities will be further magnified [41].

As populations consume more, become less self-sufficient and increasingly rely on education as the main route to socio-economic status, convergence on a single reproductive strategy is driven by a range of interrelated mechanisms that change parental investment strategies [53,54] and reproductive priorities. These include declining demand for children as economic contributors [55,56], opportunities for upward social mobility [14,57] and exposure to new cultural norms or lifestyles that promote cultural goals at the expense of reproductive ones [43,58–60]. In previous work, we found that with a critical mass of educated women in a community, less-educated women are converging on low fertility preferences [43]. Here, we examine whether multiple reproductive strategies coexist in less market-oriented communities, and if macro-level patterns in inequality are reproduced at socially and economically relevant levels of aggregation.

We test seven hypotheses designed to examine how wealth and status change in their effects on fertility, and whether this is associated with changing levels of inequality. First, if wealth and status become decoupled during market integration, then (i) they should have different effects on fertility. If reproductive stratification is driven by differences in educational capital, then (ii) educational capital should moderate how wealth influences fertility. This moderating effect should itself depend on how market-oriented the community is, and since convergence on low fertility is already underway in more highly educated communities [43], we expect (iii) reproductive strategies to vary more where farming remains a viable alternative to the labour market. Then, if converging reproductive strategies drive reductions in wealth inequality, (iv) fertility should vary less and (v) average fertility should be lower in more equal communities. Finally, if community-level inequalities reliably reproduce macro-level patterns, then (vi) wealthier, more educated and market-integrated communities should be less unequal and (vii) communities with more equal distributions of market integration and wealth should have higher educational capital.

Our data come from a randomized study of 1995 women aged 18–91 living in 22 communities (21 villages and 1 town, see electronic supplementary material, table S1) in rural Poland. Data were collected between 2009 and 2010. The area is characterized by centuries of peasant subsistence farming. It is now rapidly becoming dependent on labour-market income [61,62], following Poland's rapid transition to a market economy in the early 1990s and accession to the EU in 2004. More than 65% (*n* = 1255) of our respondents live in households partly or mainly subsisting from farming, but only approximately 4% of households exclusively farm. Income-generating strategies combine farm and off-farm work, formal and informal wage-labour, allowing us to examine both ‘traditional’ and ‘modern’ dimensions of wealth and status. These communities are undergoing late-stage DT, and fertility is declining. Nonetheless, completed fertility in our sample is dramatically higher than Polish national estimates (total fertility rate (TFR) of 1.38 in 2010, TFR of 2.16 in 1949, the most representative birth cohort), with a mean of 3.81 (s.d. 2.15) children per woman, and significant between-community variation [43].

## 2. Material and methods

### (a) Measures of wealth and status

We develop four separate, largely orthogonal measures of wealth and status: (1) ‘farming wealth’, (2) ‘non-farming wealth’, (3) women's ‘educational capital’ and (4) household ‘market integration’ (table 1; electronic supplementary material). We conceptualize educational capital and market integration as status measures because they determine access to employment opportunities and resources in market economies, as well as exposure to non-traditional norms and values. We conceptualize wealth as the resources themselves. Measures (1)–(3) were obtained from principal component analysis (PCA), allowing us to reduce a set of candidate variables to parsimonious orthogonal ‘latent’ dimensions in the data (electronic supplementary material). Market integration is a weighted composite index of occupation, occupational prestige and employment history, averaged across up to 15 householders. All four measures are continuous and standardized to have a mean of 0 and s.d. of 1.

**Table 1.** Description of the variables used to develop the four wealth and status measures.

principal component	variable name	variable description	mean	s.d.	factor loading
farming wealth	total land	total land (in hectares)	2.23	2.75	0.78
	cows	total number cows	0.79	1.83	0.76
	bulls	total number bulls	0.16	0.76	0.67
	tractor (yes; no)	household owns a tractor	0.44	0.50	0.59
	combine (yes; no)	household owns a combine harvester	0.04	0.19	0.56
non-farming wealth	computer (yes; no)	computer in the house	0.82	0.38	0.81
	internet (yes; no)	Internet connection in the house	0.74	0.44	0.81
	car (yes; no)	car in the household	0.86	0.34	0.60
	total rooms	total number of rooms	4.59	1.78	0.54
	satellite TV (yes; no)	satellite TV in the house	0.70	0.46	0.47
	total household monthly income	mean income in the house	−0.01	0.29	0.45
educational capital	mother's education	mother's highest educational level	2.53	0.96	0.83
	father's education	father's highest educational level	2.45	0.85	0.80
	respondent education	respondent's highest educational level	3.54	0.97	0.74
	mother ever worked (yes; no)	mother ever engaged in paid work	0.57	0.50	0.68
	father ever worked (yes; no)	father ever engaged in paid work	0.80	0.40	0.62
	wage income in childhood (yes; no)	parental income source in childhood	0.33	0.47	0.60
	any holiday (yes; no)	family has ever been on holiday	0.56	0.50	0.36
Cronbach's *α* = 0.57 for ‘Farming wealth’, *α* = 0.70 for ‘Non-farming wealth’, *α* = 0.82 for ‘Educational capital’					

composite variable	variables used	variable description (weight given)			
market integration	current occupation categories	farmer (1); full (2)/part-time (2)/seasonal (1) employed; unemployed/jobseeker (1); housewife/child-minder/maternity leave (1); pensioner/receiving state benefits (1); full time student (1); dependent (0)			
	occupational status categories	specialist/manager (3); qualified white-collar (3); unqualified white-collar (2); qualified blue-collar (2); unqualified blue-collar (1)			
	ever employed (yes; no)	individual had ever been employed; yes (1); no (0)			

Our measures capture detailed variation in the proxies of wealth and status. Educational capital incorporates both parental and individual education, and thus intergenerational transmission of socio-economic status, making it a good proxy for ‘embodied’ capital. Market integration incorporates occupational status and both formal and informal employment, reflecting the diversity of income-generating strategies in this population. Our measures have significant advantages over using multiple predictors measured on different scales, including better handling of inter-correlation, more parsimonious statistical modelling and direct comparability of effect sizes on fertility, both within and between communities.

We describe inequality in each of our four measures using Gini coefficients, calculated across the whole sample and disaggregated by community.

### (b) Statistical analysis

We use multi-level Poisson regression [63] to examine wealth and status associations with fertility (number of live births) in our 22 study communities, net of controls (age, age^2^, experience of under-five mortality (approx. 3% of women) and farmer status (fertility and farming wealth are higher among farmers)). We include random intercepts at the community and individual levels to account for unobserved heterogeneity at each level of analysis [63]. Twenty-three women living outside the study communities were excluded, leaving a sample size of *n* = 1972.

We allow both the slopes and intercepts of our wealth and status measures to vary by community, constituting a conservative test of the hypothesis that they are associated with fertility overall (electronic supplementary material). Completed fertility differs significantly between our study communities [43], and our interest is in how relative, rather than absolute wealth and status differentials might drive this variation. We therefore group-mean centred our predictors, transforming an individual's score into her deviation from the community mean. This removes the partial correlation between individual- and community-level effects, which is essential for contextual modelling [64]. Group-centring neither substantively affects the results (electronic supplementary material, table S4) nor creates artificial differences between communities (electronic supplement material, figures S1 and S2), and the community means are included in the model. All analyses were carried out in R v. 14.2 (electronic supplementary material).

## 3. Results

### (a) Associations between wealth, status and fertility

**Figure 1.**
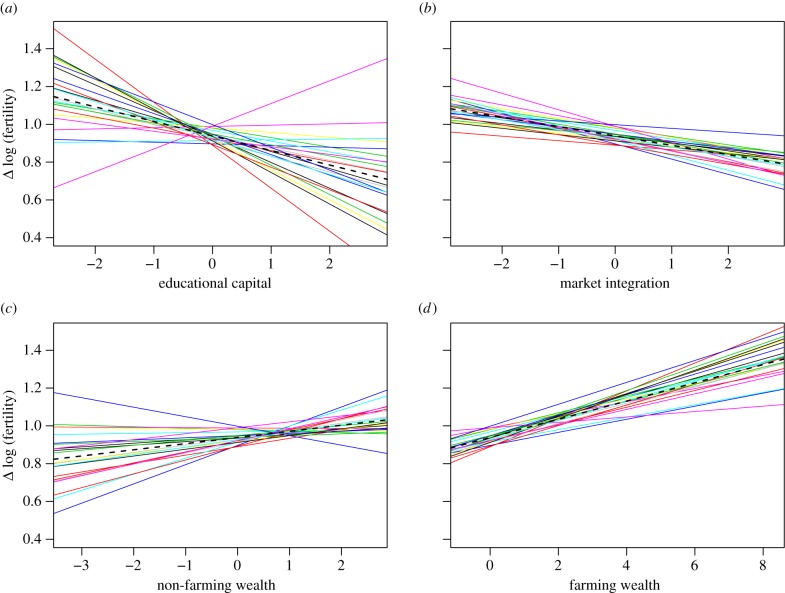
Between-community variation in the associations between fertility (using a log link) and (*a*) educational capital, (*b*) market integration, (*c*) non-farming wealth and (*d*) farming wealth. Each line represents the model-adjusted regression of each measure on fertility in each community (*n* = 1972). Dashed black lines represent the fixed effect of each measure overall. Every unit on the *x*-axis corresponds to 1 s.d. in the predictor variable; all four measures are centred on zero.

(*i*) *Wealth and status have opposing effects on fertility, and these vary by community.* Figure 1 shows that educational capital and market integration are generally negatively associated with fertility, whereas farming and non-farming wealth are generally positively associated with fertility. When only fixed effects are considered (dashed black lines), all of the associations are significant. However, allowing the slopes to vary by community reveals associations that differ and are not always significant (coloured lines).

Educational capital has the largest fixed effect on fertility. A 1 s.d. increase is associated with a 7% reduction in fertility (e*^*β*^* = 0.93, *β* = −0.08, 95% CI(*β*) [−0.14, −0.02] (*β*, regression coefficient; CI, confidence interval)). The distribution spans approximately 5.3 s.d., so women with the most educational capital were predicted to have approximately 35% (i.e. 1-e^5.3*β*^) fewer children than women with the least. But depending on the community, a 1 s.d. increase predicted anywhere from a 20% decrease (e*^*β*^* = 0.80, *β* = −0.23, 95% CI(*β*) [−0.32, −0.14]) to a 13% increase (e*^*β*^* = 1.13, *β* = 0.12, 95% CI(*β*) [0.01, 0.24]) in fertility. Thus, when considered across the range in a particular community (electronic supplementary material, figure S1), women with the most educational capital are predicted to have from approximately 60% fewer to approximately 54% more children than women with the least educational capital.

Similarly, a 1 s.d. increase in market integration is associated with a 5% decrease in fertility overall (e*^*β*^* = 0.95, *β* = −0.05, 95% CI(*β*) [−0.08, −0.01]), translating into approximately 24% lower fertility among the most market-integrated households compared with the least (the range spans approx. 5.5 s.d.). This varies across communities from a 2% (e*^*β*^* = 0.98, *β* = −0.02, 95% CI(*β*) [−0.05, 0.00]) to an 8% (e*^*β*^* = 0.92, *β* = −0.09, 95% CI(*β*) [−0.12, −0.05]) decrease in fertility or a difference of between 9 and 20% fewer children among women in the most market-integrated households compared with those in the least market-integrated ones.

A 1 s.d. increase in non-farming wealth is associated with a 3% increase in fertility overall (e*^*β*^* = 1.03, *β* = 0.03, 95% CI(*β*) [−0.01, 0.08]), and therefore, approximately 20% higher predicted fertility among the wealthiest women compared with the least wealthy (the range spans approx. 6 s.d.). This varies across communities from an 11% increase (e*^*β*^* = 1.11, *β* = 0.10, 95% CI(*β*) [0.03, 0.17]) to a 5% decrease (e*^*β*^* = 0.95, *β* = −0.05, 95% CI(*β*) [−0.10, 0.00]) in fertility, and thus a difference of between 55% more and 22% fewer children among women with the highest levels of non-farming wealth relative to the least wealthy women.

A 1 s.d. increase in farming wealth is associated with a 5% increase in fertility overall (e*^*β*^* = 1.05, *β* = 0.05, 95% CI(*β*) [0.02, 0.08]) and therefore approximately 65% higher predicted fertility among the wealthiest women compared with the least wealthy women (the range spans approx. 10 s.d.). This varies across communities from a 1% (e*^*β*^* = 1.01, *β* = 0.01, 95% CI(*β*) [−0.01, 0.03]) to an 8% (e*^*β*^* = 1.08, *β* = 0.07, 95% CI(*β*) [0.06, 0.09]) increase in fertility. The most traditionally wealthy women are predicted to have between 5 and 34% more children than the least wealthy women in their community.

**Figure 2.**
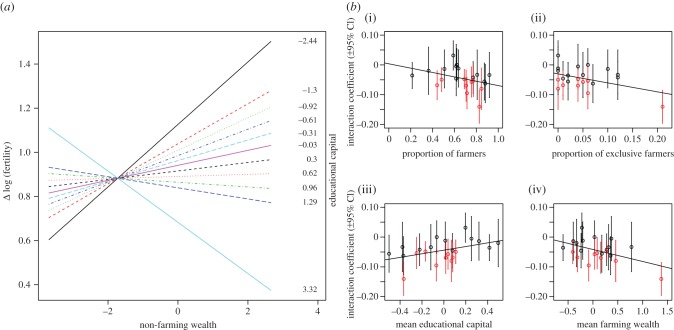
(*a*) The (fixed effect) interaction between non-farming wealth and fertility, for deciles on the scale of educational capital. The top (solid black) line illustrates the positive association between wealth and fertility for women with the lowest educational capital. The bottom (light blue) line illustrates the negative association between wealth and fertility for women with the highest educational capital. Each unit on the *x*-axis corresponds to 1 s.d. in non-farming wealth. (*b*) The interaction is stronger (i.e. more negative) in communities with more (i) farmers and (ii) exclusive farmers, and in communities with (iii) low mean educational capital and (iv) high mean farming wealth. The interaction is not always significant: red points indicate where the 95% CIs do not include zero.

These results show that the magnitude and sometimes the direction of these effects on fertility depend on the local context in which women reproduce, and imply non-trivial fertility differentials. Nonetheless, a significant interaction between non-farming wealth and educational capital (figure 2*a*) means that these effects should not be understood in isolation.

(*ii*) *Educational capital modifies the effect of non-farming wealth on fertility.* Figure 2*a* shows that overall, non-farming wealth is positively associated with fertility when educational capital is low (top black line), but negatively associated with fertility when educational capital is high (bottom blue line). A 1 s.d. increase in a woman's educational capital is associated with a 4% decrease in the magnitude of the positive relationship between non-farming wealth and fertility (e*^*β*^* = 0.96, *β* = −0.04, 95% CI(*β*) [−0.08, −0.01]). Women with higher educational capital are therefore predicted to *reduce* fertility with increasing wealth, whereas women with lower educational capital are predicted to *increase* fertility with increasing wealth. However, the interaction itself varies by community and is not always significant (figure 2*b*). A 1 s.d. increase in educational capital is associated with anywhere from a 3% increase (e*^*β*^* = 1.03, *β* = 0.03, 95% CI(*β*) [−0.02, 0.08]) to a 13% decrease (e*^*β*^* = 0.87, *β* = −0.14, 95% CI(*β*) [−0.20, −0.09]) in the magnitude of the positive relationship between non-farming wealth and fertility.

(*iii*) *Diverging reproductive strategies are more evident where farming is viable.* Figure 2*b* shows that the interaction above tends to be stronger (i.e. more negative) and more often statistically significant within communities with a high proportion of farmers (usually more than 60%, Pearson's *R* = −0.38, *p* = 0.093) and exclusive farmers (*R* = −0.44, *p* = 0.043), and in communities with higher mean farming wealth (*R* = −0.47, *p* = 0.026) and lower mean educational capital (*R* = 0.42, *p* = 0.050). Thus, where farming livelihoods are viable, reproductive strategies are more variable. The interaction is not related to community sample size (*R* = 0.017, *p* = 0.939), nor to population density (*R* = 0.051, *p* = 0.822).

We examined whether educational capital drives postponement of reproduction, either as a strategy to increase wealth among highly educated women [35], or because of a trade-off between education and early childbearing [65]. We do not find support for either of these hypotheses. Women in the top quartile of educational capital do not have significantly later ages at first birth (AFB), either when pooling all age-groups (*F* = 1.21, *p* = 0.304, mean AFB = 23.76 ± 3.83), or considering only post-reproductive women (*F* = 1.43, *p* = 0.232), and the top quartiles are not significantly different across communities (*F* = 1.09, *p* = 0.297). Women in the top quartile of educational capital do not differ in levels of non-farming wealth, either across the sample (*F* = 0.45, *p* = 0.720) or across communities (*F* = 1.13, *p* = 0.287), although they exhibit significantly less variance in wealth (Browne–Forsythe *F* = 24.81, *p* < 0.001) and are substantially more market-integrated (*F* = 77.69, *p* < 0.001). Instead, post-reproductive women in the top quartile of educational capital have significantly shorter reproductive spans (*F* = 13.35, *p* < 0.001), indicating that later fertility regulation within marriage, rather than postponement of early reproduction, drives the patterns in our data. Neither the main effect of educational capital, nor its interaction with non-farming wealth, is driven by the inclusion of childless women in our sample (electronic supplement material, tables S5–S6), contrary to what has been found in other studies [26,27].

### (b) Wealth and status inequality

**Figure 3.**
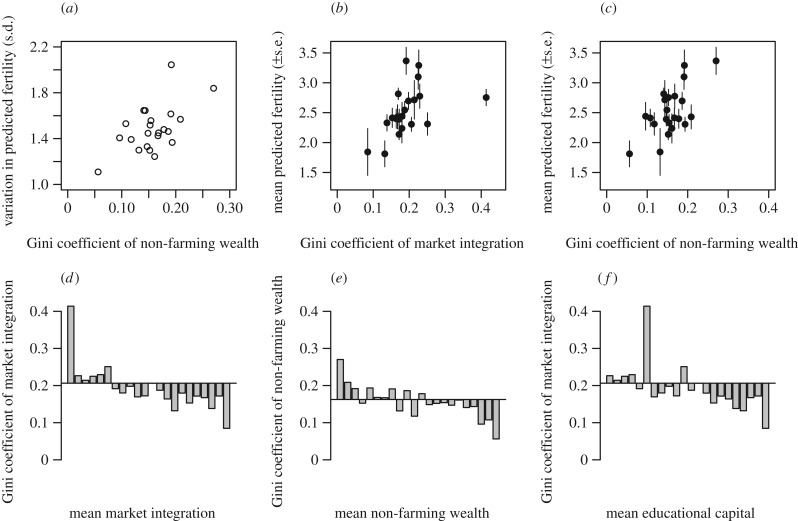
(*a*) Variation in predicted fertility (measured in s.d.) in lower in communities with lower inequality in non-farming wealth. Mean predicted fertility in the community (±s.e. in the prediction) is also lower in communities with lower inequality in (*b*) market integration and (*c*) non-farming wealth. Inequality in (*d*) market integration and (*e*) non-farming wealth declines as the mean in the community increases. (*f*) Mean educational capital is higher in communities where inequality in market integration is lower. Each bar in (*d*)–(*f*) represents a Gini coefficient in a particular community, shown as a deviation from the overall Gini coefficient for that measure (horizontal line). Communities are ordered from left to right in terms of increasing mean (*d*) market integration (*e*) non-farming wealth and (*f*) educational capital.

Overall, inequality across the sample is relatively low, with Gini coefficients of 0.23 for educational capital, 0.21 for market integration, and 0.16 for non-farming wealth. Farming wealth has a higher Gini coefficient of 0.42 (measuring only farmers). However, inequality varies substantially across communities. Gini coefficients for educational capital vary from 0.13 to 0.26, indicating twice the amount of inequality in some communities compared with others. Gini coefficients for market integration vary from 0.08 to 0.41, and those for non-farming wealth vary from 0.06 to 0.27, in both cases indicating up to five times more inequality in some communities compared with others. Coefficients for farming wealth vary from 0.05 to 0.56, indicating ten times the amount of inequality in the most unequal community compared with the most equal one. Gini coefficients for different measures are not significantly inter-correlated (electronic supplementary material, table S2c), so inequality on one dimension does not imply inequality on others. Inequality is also unrelated to population density or sample size (electronic supplementary material, table S2d).

(*iv–v*) *Fertility is higher and more varied in unequal communities.* We examined correlations between community-level Gini coefficients and the community-averaged fitted scores from the multi-level model (adjusted means and s.d. of predicted fertility). Figure 3*a* shows that there is significantly less variation in predicted fertility (measured in s.d.) in communities with lower inequality in non-farming wealth (*R* = 0.58, *p* = 0.004), but variation in fertility is not related to other forms of inequality. Figure 3*b*,*c* show respectively that mean predicted fertility is also significantly lower in communities with lower inequality in both market integration (*R* = 0.47, *p* = 0.027) and non-farming wealth (*R* = 0.65, *p* = 0.001), but is not related to inequality in educational capital. Declining inequality in wealth and market integration is therefore related to convergence on low fertility strategies, but not all kinds of inequality are equally important.

(*vi–vii*) *Community-level inequality reproduces macro-level patterns.* Finally, community-level inequalities reproduce macro-level patterns for some measures. Figure 3*d*,*e* show respectively a clear decline in inequality in both market integration (*R* = −0.85, *p* < 0.001) and non-farming wealth (*R* = −0.87, *p* < 0.001) in communities where the mean is higher. However, inequality in educational capital and farming wealth is not related to the means of those measures. Figure 3*f* also confirms that in communities where inequality in market integration is lower, mean educational capital is significantly higher (*R* = −0.57, *p* = 0.005, [the outlier in figure 3*f* is the community with the smallest sample size of *n* = 11]). This last result is not driven by in-migration of highly educated people into more market-integrated communities. The proportion of migrants in the community is unrelated to inequality in market integration (*R* = 0.143, *p* = 0.526) and migrants are not more highly educated than non-migrants (*t* = 0.122, *p* = 0.903).

## 4. Discussion

Transitions from hunting and gathering to subsistence agriculture appear typified by a diversification in the types of wealth and status that are inherited, and by increasing reliance on ‘extrasomatic’ over ‘embodied’ capital [20,21]. By contrast, contemporary transitions to market economies seem typified by the re-emergence of embodied forms of capital as central to socio-economic [38], but not necessarily reproductive success [66,67]. Demographic transitions can therefore be conceptualized as transitions in the nature and effects of wealth and status. The diversification of reproductive strategies that these changes allow may drive increases in inequality. Convergence on low fertility may then temporarily reduce these inequalities. We demonstrate connections between the two broad reversals that tend to characterize contemporary DTs to low fertility, at a level of aggregation that captures the social and economic contexts people actually live in. Our results provide a rarely available insight into the changing dynamics of wealth, status and inequality in one of the few European regions where this change is still ongoing.

We find that wealth and status have different effects on fertility, but the associations vary by community, and they interact in certain contexts to produce patterns typical of both pre- and post-DT populations. In this mid-transition context, wealthy women with higher levels of educational capital—a form of status that itself emerges and becomes important during economic development—have fewer children, whereas wealthy women with lower educational capital have more children. These are not simply differences in fertility outcomes between high- and low-status individuals, but imply different reproductive strategies, i.e. the use of resources to maximize different fitness currencies [68]. These different reproductive strategies coexist in communities where farming remains an important livelihood.

Women in the top quartile of educational capital were neither more likely to postpone the start of their reproductive careers, nor more materially wealthy, than their counterparts in lower quartiles, either across communities or as a whole. So fertility reduction does not appear to be an unambiguous strategy for acquiring material advantages early in life, although women in the top quartile of educational capital do exhibit less variance in wealth. Rather, fertility reduction appears driven by reproductive choices after the onset of childbearing. This may have more to do with changing preferences over the course of the reproductive career than with fundamental energetic trade-offs at the onset of reproduction: it may also be related to time constraints owing to workforce participation at later life stages. These results contrast with recent evidence that low fertility, high educated women have wealth advantages [35], and that early childbearing impedes educational attainment [65].

In line with macro-level and some historical evidence [5,14,39], fertility decline in this population is associated with declining inequality in wealth and market integration, but not with declining inequality in educational capital. This is broadly consistent with a ‘Kuznetz curve’ such that inequality has an inverted U-shaped relationship with average income [4]. This could be driven by the rapid abandonment of farming [61], but is also consistent with the diffusion of knowledge and values that alter reproductive preferences in a way that may causally influence economic behaviour [8,43,58]. Our analyses certainly support the assumption that more equality in market integration is related to the accumulation of educational capital [5,39]. So why was inequality in educational capital unimportant for predicting fertility decline? The answer lies partly in the fact that market engagement in this population is not dependent on high levels of education, as informal and migrant wage-labour, and to a lesser extent, seasonal cash cropping, can generate significant financial returns for less-educated households. This explains why women with the highest educational capital, while more market integrated, were not necessarily wealthier. But also, as we have previously shown, less-educated women appear to adopt the reproductive strategies of their peers, given a critical mass of highly educated women in the community, so individual-level variation in education does not necessarily track variation in fertility [43].

Until recently, this largely self-sufficient economy was reliant on cooperative farm work and childcare [61,62]. With the diminishment of farming [61], reproductive strategies are converging on the solution we now see in the majority of the world's populations [43]. Our results capture reproductive stratification alongside new kinds of status stratification, but the effects of wealth and status on fertility should not be considered in isolation. We expect similar interactions to exist in other transitioning populations, and cross-cultural work could establish whether they are a general feature of socio-economic stratification. A simple dichotomy between wealth (understood as resources) and status (understood as access to resources) is useful for generalizing across populations and for generating causal hypotheses. We have argued that status differentials drive the changing relationship between wealth and fertility, but wealth and status are not independent; there will undoubtedly be important feedback between them. However, diverging reproductive strategies that magnify differentials between farmers and non-farmers may drive increases in wealth inequality. This diversification, and its subsequent convergence, may be a central mechanism in the changing inequalities that accompany the later stages of contemporary DTs, as economic growth takes off.

## Supplementary Material

Electronic Supplementary Materials

## Data accessibility

For access to the data used in this paper, contact H.C.: heidi.colleran@iast.fr.
