# Supplementary material for: Fertility decline and the changing dynamics of wealth, status and inequality
Source: Proc Biol Sci. 2015 May 7;282(1806):20150287. doi: 10.1098/rspb.2015.0287 (PMC4426630; doi:10.1098/rspb.2015.0287)
Supplement: Electronic Supplementary Materials [file rspb20150287supp1.pdf]

Electronic Supplementary Materials

"*Fertility decline and the changing dynamics of wealth, status and inequality*"

Heidi Colleran, Grazyna Jasienska, Ilona Nenko, Andrzej Galbarczyk and Ruth Mace

Contents:

Supplementary Discussion

Supplementary Tables S1-S7

Supplementary Figures S1-S2

Supplementary References

Supplementary discussion

1. Sampling and data collection

Our sample of communities (21 villages and one town) was randomly drawn from four neighbouring municipalities containing a total of 34 potential study populations. All 22 of our sampled communities agreed to participate. We did not have access to a list of names or a map of households, so within each community every third house was sampled, with every adult woman (≥18 years) present in the house invited to take part in the survey. This strategy was further stratified in the town, by randomly selecting streets from a list obtained from the local government and approaching every third house/apartment on that particular street. We returned to houses that were unoccupied at the time of selection on up to three occasions. All consenting adult women were interviewed. All eligible women who declined to take part were noted as non-responders, as were women ineligible to take part due to age or illness. 52.4% to 89.4% of respondents who were approached in any village agreed to an interview, and the average response rate for the entire sample was 75% (total interviews ÷ [total responders + non-responders]). Response rates for each of the communities as well as sample sizes and other community descriptors are given in Table S1.

Our sampling strategy means that important variables such as age are approximately normally distributed in all groups. The sample should not be considered representative of the country as a whole, but of the wider rural population of this particular region. Our samples are representative of the villages women inhabit and the aggregates we use are therefore appropriate to our research question.

Survey data were collected by semi-structured interview in Polish, conducted during the pilot study by HC, IN and AG, and by HC and trained local field assistants during the main data collection. During the pilot study (2009), GJ, IN and AG were funded by the National Science Center (Grant N404 273 440), Ideas Plus (Grant IdP2011 000161) and the Foundation for Polish Science, but these did not fund the study.

	Population descriptors						Basic demographics					Access to amenities		
Community ID	Population size	Population density	Sample size	Response rate (%)	Distance to town (km)	Total area (km^2)	Completed fertility	Mean age at first birth	Prop. living in natal village	Prop. farmers	Prop. u-5 mortality	Primary school	Secondary school	Health center
1	644	61.7	122	85.9	13.2	10.4	4.02	23.10	0.56	0.77	0.05	+	-	-
2	3890	183.7	129	79.6	2.6	21.2	4.06	23.32	0.67	0.59	0.03	+	+	-
3	3194	178.1	102	73.4	9.0	17.9	3.84	23.38	0.71	0.71	0.04	+	+	-
4	1988	126.4	135	89.4	3.6	15.7	3.06	23.90	0.67	0.76	0.03	-	+	-
5	689	102.2	59	83.1	6.9	6.7	4.75	22.61	0.54	0.88	0.04	-	-	-
6	1501	149.3	105	87.5	6.7	10.1	3.67	22.94	0.59	0.71	0.04	-	+	-
7	275	97.0	26	83.9	2.8	2.8	4.64	24.50	0.50	0.85	0.00	-	-	-
8	1368	176.1	85	73.9	3.8	7.8	3.65	24.31	0.49	0.48	0.02	+	-	-
9	227	81.0	11	52.4	4.8	2.8	3.80	26.75	0.64	0.36	0.00	-	-	-
10	780	254.9	78	69.0	1.5	3.1	4.00	24.66	0.50	0.62	0.07	-	-	-
11	740	181.4	61	58.7	1.8	4.1	3.83	23.48	0.59	0.51	0.05	-	-	-
12	14,900	784.2	206	80.8	0.0	19.0	3.41	24.12	0.58	0.21	0.02	+	+	+
13	3150	157.6	136	63.8	11.2	20.0	3.03	24.44	0.64	0.44	0.03	+	+	+
14	566	91.1	52	70.3	12.2	6.2	3.35	24.26	0.59	0.83	0.09	+	-	-
15	623	125.1	26	57.8	15.0	5.0	3.56	25.07	0.58	0.62	0.00	-	-	-
16	352	53.5	37	74.0	14.7	6.6	3.45	25.14	0.51	0.92	0.03	-	-	-
17	1112	116.2	108	87.8	11.5	9.6	3.53	23.87	0.66	0.62	0.04	+	+	+
18	613	66.4	55	69.6	13.4	9.2	3.56	22.90	0.67	0.64	0.02	+	-	-
19	493	22.7	63	86.3	13.4	21.7	4.72	24.75	0.56	0.87	0.07	+	-	-
20	6237	109.4	284	69.4	7.2	57.0	4.64	23.40	0.72	0.69	0.02	+	+	+
21	353	59.5	26	72.2	10.6	5.9	3.92	22.19	0.46	0.81	0.04	-	-	-
22	721	94.8	66	82.5	7.9	7.6	4.52	23.45	0.55	0.77	0.03	+	-	-
Grand mean	2019	148.8	89.6	75.1	7.9	12.3	3.86	23.93	0.59	0.67	0.03			

Table S1 Descriptive overview of the study populations. Population descriptors, basic demographic characteristics and access to local amenities are given for each study group separately, with the grand mean given at the bottom of the table. Villages and town are not identified by name, only by ID number, which indicates the order in which they were sampled

2. Variable construction

Principal Component Analysis (PCA) produces weighted linear combinations (components) of inter-correlated variables, explaining as much variance as possible using as few dimensions as possible. Each individual receives a score on a particular dimension based on the weighted linear combination of all the variables used in the analysis. This helps to avoid both multicollinearity and loss of dimensionality by reducing a large set of inter-correlated variables to a smaller number of uncorrelated (or minimally correlated) dimensions. PCA provides an excellent and increasingly popular set of tools for creating wealth and status measures [1–3].

A total of 25 different variables were initially considered in constructing the three variables: non-farming and farming wealth and educational capital. Since farming-related variables (e.g. ownership of a tractor) apply to farmers only, whereas all other variables (e.g. education, cars or computers) apply equally to all women in the sample, the PCA on farming resources was run separately from that on the other variables. This avoids biasing the PCA due to high variance between farmers and non-farmers (i.e. the fact that non-farmers necessarily have low levels of farming resources). All other non-farming variables were analysed together.

We removed variables from the two PCAs on the basis that they either: (1) did not correlate significantly with any other variables (a prerequisite for PCA); (2) showed poor communalities (i.e. did not share variance with the other variables – shared variation is needed to create meaningful constructs out of multiple variables); or (3) showed poor scalability with other variables, as measured using Cronbach's α statistic, which describes the inter-item scale reliability of the variables in the analysis. Each of the three factors produced using PCA contained at least 5 items with high factor loadings (~0.50 or higher), so each dimension can be considered a stable construct [2]. Missing values were imputed with the mean, though there was not much missing data. These three continuous measures have a mean of 0 and a standard deviation of ~1. Given the large sample size (total n = 1,972), all the factor loadings can be considered statistically significant at an alpha level of 0.01 (two-tailed). Variable communalities, indicating the extent to which the items shared variance with other items in the analysis, were relatively high, with an average of 47% shared variance among the variables (see Table 1 in the main text). This is consistent with what would typically be expected from social science data [2].

Although we originally considered variables relating to 'relational wealth' (see [4,5]), in the form of number of contacts (both kin and non-kin) in the community, these variables did not constitute a separate dimension in any PCA, nor did they correlate with most of the variables used to measure non-farming wealth. They also did not scale well with other variables and were removed from the final analysis (Cronbach's α for the scale of educational capital was reduced from 0.82 to 0.02 with the addition of 'relational wealth' variables). This suggests that relational wealth does not constitute a separate dimension of wealth in this population.

Note that we do not examine a separate measure of husband's educational capital in the multilevel analysis, for a number of reasons. First, we wish to include unmarried women who may have had children in the analysis so including data on husbands would restrict the sample to married women only. Second, there is high assortativity in educational capital between husbands and wives in this population (Pearson's R = 0.69, $p < 0.001$) and we do not wish to introduce collinearity into the models. However it should not be assumed that we do not take into account husband's wealth and status in this analysis; where women are married,

their husband's wealth and status characteristics are reflected in non-farming and farming wealth, as well as household market integration, which takes into account everybody in the household.

(a) Inter-correlation of individual level wealth and status measures

	Market integration	p-value	Non-farming wealth	p-value	Farming wealth	p-value
Educational capital	0.330	0.000	-0.003	0.897	-0.248	0.000
Market integration	.	.	0.406	0.000	-0.301	0.000
Non-farming wealth	.	.	.	.	0.023	0.303

(b) Inter-correlation of community means of wealth and status

	Market integration	p-value	Non-farming wealth	p-value	Farming wealth	p-value
Educational capital	0.864	0.000	0.236	0.289	-0.480	0.024
Market integration	.	.	0.305	0.168	-0.340	0.127
Non-farming wealth	.	.	.	.	0.210	0.342

(c) Inter-correlation of Gini coefficients

	Gini Market integration	p-value	Gini Non-farming wealth	p-value	Gini Farming wealth	p-value
Gini Educational capital	-0.390	0.072	0.064	0.787	0.160	0.461
Gini Market integration	.	.	0.150	0.491	0.180	0.427
Gini Non-farming wealth	.	.	.	.	0.330	0.129

(d) Inequality is not correlated with population density or sample size

	(i) Population density		(ii) Sample size	
	Pearson's R	p value	Pearson's R	p value
Gini Educational capital	-0.140	0.535	-0.034	0.880
Gini Market integration	-0.219	0.327	0.073	0.745
Gini Non-farming wealth	0.021	0.925	0.059	0.794
Gini Farming wealth	0.303	0.170	0.401	0.064

Table S2 Inter-correlations between wealth and status at (a) the individual level, (b) the community level, (c) inter-correlations between the community level Gini coefficients and (d) correlations between the Gini coefficients and (i) population density and (ii) sample size. All tests show Pearson's correlation coefficients and exact p-values.

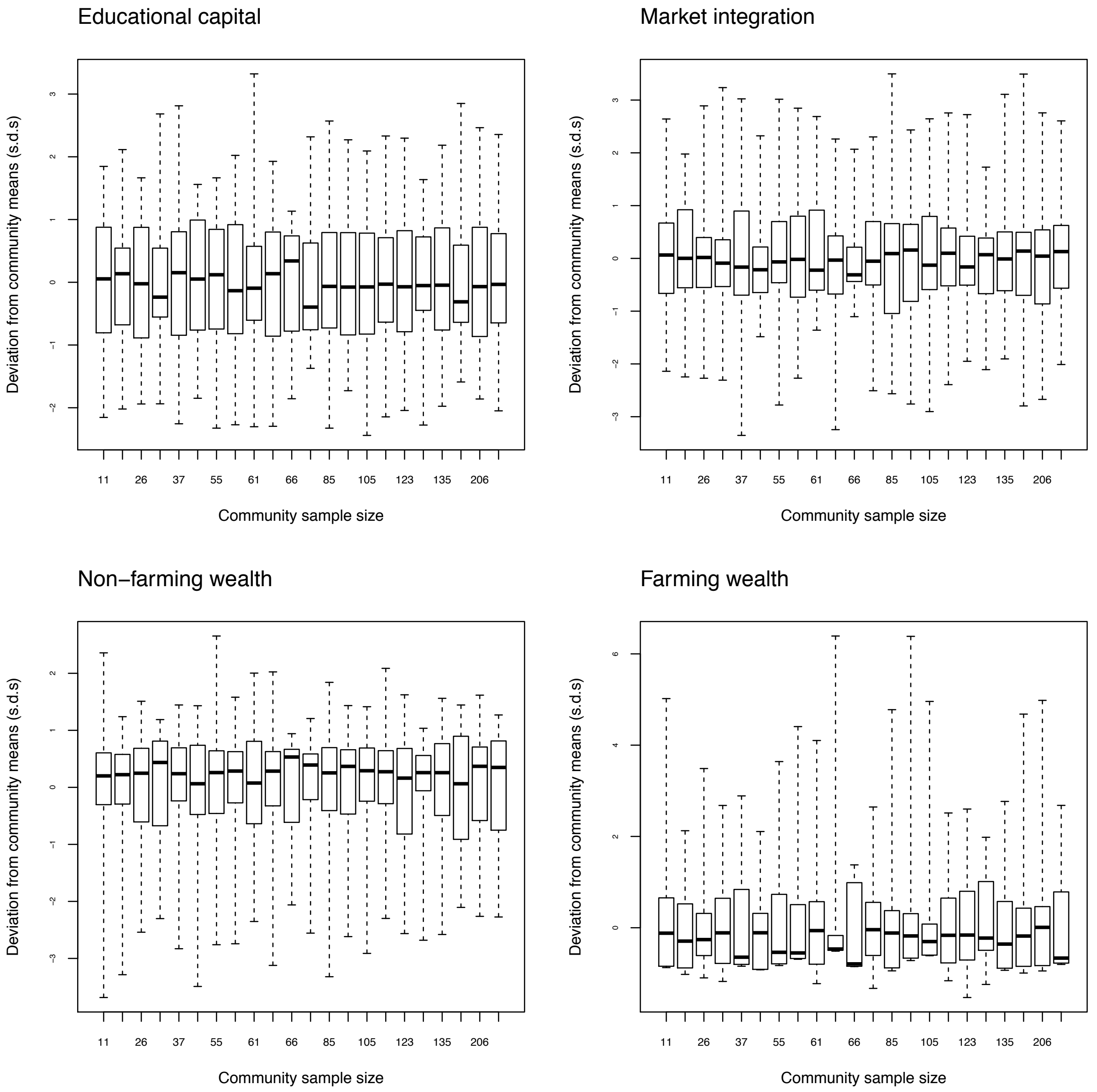

Figure S1. Group mean centered individual level data. Each panel shows the range of deviations (in s.d.s) from the mean of each community for a particular measure, given in order of increasing sample size from left to right. All individual scores are centered on zero since the mean is removed. Box and whisker plots show the full distribution of deviations (given in s.d.s), medians (black dashes), quartiles (25% on each side of the median), and minimum and maximum values in each of the 22 communities.

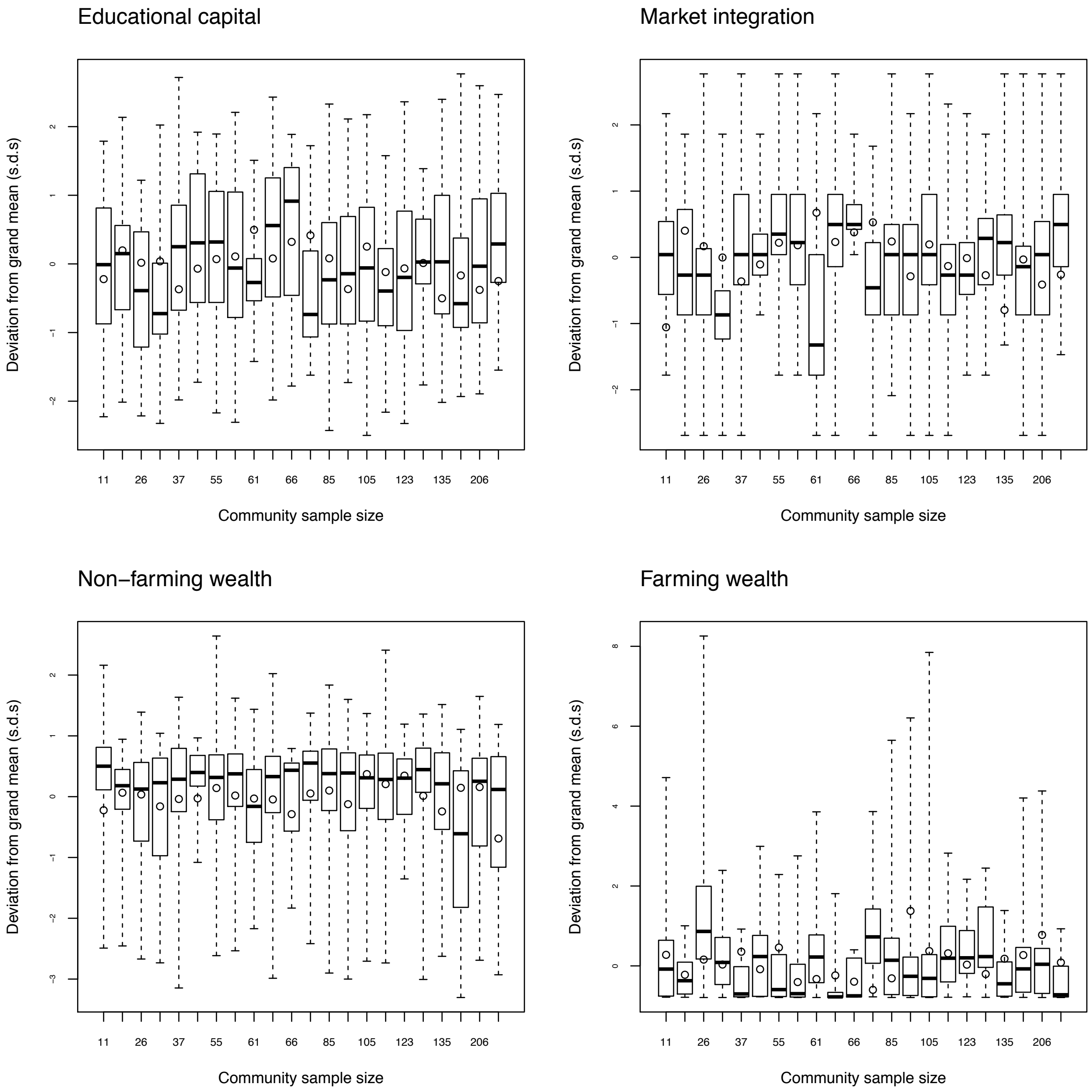

Figure S2. Grand mean centered data (the grand mean is zero). Each panel shows box and whisker plots of the untransformed distributions of wealth and status in the 22 communities (in s.d.s) given in order of increasing sample size from left to right. Boxplots give median (black dashes), quartiles (25% on each side of the median), minimum and maximum values in each of the 22 communities, with empty circles representing the group mean.

3. Data analysis

To avoid boundary problems associated with small variance parameters and a small sample of groups, including incorrect or underestimated uncertainty in the model parameters and covariance matrices, we used maximum penalized likelihood (MPL) [17-19], with the Laplace approximation, to estimate our model. MPL uses 'weakly informative' or 'diffuse' priors to obtain Bayesian modal estimates on the parameters in the model. This ensures that the variance component estimates remain off the boundary of the feasible parameter space (zero), but with weak enough priors so that inferences remain consistent with the data [17-19]. This method extends standard multilevel modelling techniques without requiring simulation as in fully Bayesian analysis (which obtains posterior mean estimates) and outperforms standard maximum likelihood methods [6,7]. All our analysis was carried out in R, version 14.2 [8], using the 'lme4'[9], 'arm'[10], 'blme'[11], "languageR"[12] and "reldist" [13]packages.

We allowed the slopes of all four of our predictors to vary by community. This allows the standard errors around the main effect estimates to be wider, reducing the chances of finding significant fixed effects where none exist. Our tests of the fixed effects of wealth and status on fertility are therefore conservative.

Our group centering strategy is driven by the theoretical question we are asking, i.e. whether relative effects of wealth and status matter for predicting fertility. Group-centered variables, because they are interpreted in a different way in statistical models, are not expected to produce exactly the same results as the equivalent, un-centered variables [14]. This is because the former is measured as a deviation from the group mean, and the latter as a deviation from the grand mean. Nonetheless the centering strategy has no substantive effect on the results, it simply partitions the variance more cleanly into within and between community components, although of course it alters the interpretation of within-community coefficients (i.e. they are relative rather than absolute effects). Community means are included in the models we report in the main mamuscript.

	(A) Null model						(B) Fixed effects - excluding interaction								(C) Fixed effects - including interaction							
**Predictor**	**β**	**s.e.**	**exp(β)**	**z-value**	**Pr(<|z|)**		**β**	**s.e.**	**exp(β)**	**95% CI (β)**		**z-value**	**Pr(<|z|)**		**β**	**s.e.**	**exp(β)**	**95% CI (β)**		**z-value**	**Pr(<|z|)**	
Intercept	0.78	0.03	2.19	29.64	0.000	***	0.93	0.04	2.55	0.85	1.02	22.38	0.000	***	0.93	0.04	2.52	0.84	1.01	22.34	0.000	***
Controls																						
Relative age							0.04	0.00	1.04	0.04	0.04	24.40	0.000	***	0.04	0.00	1.04	0.03	0.04	23.98	0.000	***
Relative age^2							0.00	0.00	1.00	0.00	0.00	-18.07	0.000	***	0.00	0.00	1.00	0.00	0.00	-17.73	0.000	***
Experienced under 5 mortality (ref = no)							0.31	0.06	1.36	0.19	0.43	5.06	0.000	***	0.31	0.06	1.36	0.19	0.43	5.06	0.000	***
Farmer (ref = no)							0.09	0.04	1.09	0.01	0.17	2.30	0.021	*	0.09	0.04	1.10	0.02	0.17	2.45	0.014	*
Status & Wealth																						
Relative Educational capital							-0.08	0.02	0.93	-0.12	-0.03	-3.45	0.001	***	-0.08	0.02	0.92	-0.13	-0.04	-3.64	0.000	***
Relative Market integration							-0.04	0.02	0.96	-0.08	-0.01	-2.54	0.011	*	-0.04	0.02	0.96	-0.07	-0.01	-2.43	0.015	*
Relative Non-farming wealth							0.04	0.02	1.04	0.01	0.08	2.64	0.008	**	0.01	0.02	1.01	-0.02	0.05	0.74	0.460	
Relative Farming wealth							0.05	0.02	1.05	0.01	0.08	2.84	0.005	**	0.04	0.02	1.04	0.01	0.07	2.71	0.007	**
Relative Educational capital*RelativeNon-farming wealth															-0.05	0.02	0.95	-0.08	-0.02	-3.28	0.001	**
Group level predictors																						
Mean Educational capital																						
Mean Market integration																						
Mean Non-farming wealth																						
Mean Farming wealth																						
Variance components																						
Between-community intercept variance	0.006						0.013								0.012							
Between-individual intercept variance	0.298						0.001								0.001							
Educational capital slope variance																						
Market integration slope variance																						
Non-farming wealth slope variance																						
Farming wealth slope variance																						
Educational capital*Non-farming wealth slope variance																						
Model fit and summary																						
Sample size	1972						1972								1972							
Log likelihood	-4062.29						-3336.20								-3331.00							
DIC	8124.58						6672.40								6661.96							
Δ DIC compared to previous	.						-1452.18								-10.44							

p < 0.05*, p < 0.005**, p < 0.001***

Table S3. Breakdown of the final multilevel Poisson regression model predicting individual fertility (n = 1,972) across the 22 communities, using group-mean centered variables (A) Null model, (B) Fixed effects only, (C) Fixed effects including the fixed interaction between non-farming wealth and educational capital. DIC values show that in every case the subsequent model is a better fit to the data.

	(D) Varying slopes								(E) Community means without varying slopes								(F) Community means with varying slopes							
**Predictor**	**β**	**s.e.**	**exp(β)**	**95% CI (β)**		**z-value**	**Pr(<|z|)**		**β**	**s.e.**	**exp(β)**	**95% CI (β)**		**z-value**	**Pr(<|z|)**		**β**	**s.e.**	**exp(β)**	**95% CI (β)**		**z-value**	**Pr(<|z|)**	
Intercept	0.93	0.04	2.54	0.84	1.02	20.88	0.000	***	0.93	0.03	2.54	0.87	1.00	27.84	0.000	***	0.94	0.03	2.56	0.87	1.00	27.70	0.000	***
Controls																								
Relative age	0.04	0.00	1.04	0.04	0.04	24.10	0.000	***	0.04	0.00	1.04	0.03	0.04	23.91	0.000	***	0.04	0.00	1.04	0.04	0.04	24.07	0.000	***
Relative age[2]	0.00	0.00	1.00	0.00	0.00	-18.19	0.000	***	0.00	0.00	1.00	0.00	0.00	-17.64	0.000	***	0.00	0.00	1.00	0.00	0.00	-18.08	0.000	***
Experienced under 5 mortality (ref = no)	0.32	0.06	1.38	0.20	0.44	5.24	0.000	***	0.30	0.06	1.35	0.18	0.42	4.90	0.000	***	0.30	0.06	1.35	0.18	0.42	4.91	0.000	***
Farmer (ref = no)	0.08	0.04	1.09	0.01	0.16	2.16	0.031	*	0.07	0.04	1.08	0.00	0.15	1.92	0.055	.	0.07	0.04	1.07	-0.01	0.15	1.78	0.075	.
Status & Wealth																								
Relative Educational capital	-0.08	0.03	0.93	-0.14	-0.02	-2.46	0.014	*	-0.08	0.02	0.92	-0.13	-0.04	-3.67	0.000	***	-0.08	0.03	0.93	-0.14	-0.02	-2.47	0.014	*
Relative Market integration	-0.05	0.02	0.95	-0.08	-0.01	-2.56	0.011	*	0.01	0.02	1.01	-0.08	-0.01	0.74	0.461		-0.05	0.02	0.95	-0.08	-0.01	-2.78	0.006	**
Relative Non-farming wealth	0.03	0.02	1.03	-0.01	0.07	1.35	0.178		-0.04	0.02	0.96	-0.02	0.05	-2.48	0.013	*	0.03	0.02	1.03	-0.01	0.08	1.43	0.152	
Relative Farming wealth	0.05	0.02	1.05	0.01	0.08	2.81	0.005	**	0.05	0.02	1.05	0.02	0.08	2.96	0.003	**	0.05	0.02	1.05	0.02	0.08	2.91	0.004	**
Relative Educational capital*RelativeNon-farming wealth	-0.05	0.02	0.95	-0.08	-0.01	-2.49	0.013	*	-0.05	0.02	0.95	-0.08	-0.02	-3.26	0.001	**	-0.04	0.02	0.96	-0.08	-0.01	-2.36	0.018	*
Group level predictors																								
Mean Educational capital									-0.17	0.04	0.85	-0.24	-0.09	-4.20	0.000	***	-0.17	0.04	0.85	-0.24	-0.09	-4.32	0.000	***
Mean Market integration									0.05	0.03	1.06	-0.01	0.12	1.66	0.097	.	0.05	0.03	1.05	-0.01	0.11	1.68	0.093	.
Mean Non-farming wealth									-0.03	0.02	0.97	-0.06	0.01	-1.65	0.100	.	-0.04	0.02	0.96	-0.07	-0.01	-2.28	0.023	*
Mean Farming wealth									-0.04	0.03	0.96	-0.09	0.01	-1.57	0.117		-0.05	0.03	0.95	-0.10	0.00	-1.78	0.076	.
Variance components																								
Between-community intercept variance	0.018								0.002								0.002							
Between-individual intercept variance	0.000								0.002								0.000							
Educational capital slope variance	0.009																0.009							
Market integration slope variance	0.001																0.001							
Non-farming wealth slope variance	0.002																0.003							
Farming wealth slope variance	0.000																0.000							
Educational capital*Non-farming wealth slope variance	0.002																0.002							
Model fit and summary																								
Sample size	1972								1972								1972							
Log likelihood	-3319.20								-3319.60								-3307.40							
DIC	6638.43								6639.21								6614.79							
Δ DIC compared to previous	-23.53								0.79								-24.42							

$p < 0.05$*, $p < 0.005$**, $p < 0.001$***

Table S3 continued. Breakdown of the final multilevel Poisson regression model predicting individual fertility (n = 1,972) across the 22 communities. (D) Varying slopes without community means, (E) Community means without varying the slopes, (F) Community means and varying slopes. DIC values show that models with varying slopes fit the data better than do models without varying slopes.

	(A) Fixed effects - excluding interaction								(B) Fixed effects - including interaction							
**Predictor**	**β**	**s.e.**	**exp(β)**	**95% CI (β)**		**z-value**	**Pr(<|z|)**		**β**	**s.e.**	**exp(β)**	**95% CI (β)**		**z-value**	**Pr(<|z|)**	
Intercept	0.94	0.04	2.55	0.86	1.01	24.90	0.000	***	0.93	0.04	2.53	0.86	1.00	24.77	0.000	***
Controls																
Age	0.04	0.00	1.04	0.04	0.04	25.01	0.000	***	0.04	0.00	1.04	0.04	0.04	24.57	0.000	***
Age^2	0.00	0.00	1.00	0.00	0.00	-19.14	0.000	***	0.00	0.00	1.00	0.00	0.00	-18.77	0.000	***
Experienced under 5 mortality (ref = no)	0.32	0.06	1.37	0.20	0.44	5.23	0.000	***	0.32	0.06	1.37	0.20	0.44	5.24	0.000	***
Farmer (ref = no)	0.09	0.04	1.10	0.02	0.17	2.53	0.011	*	0.10	0.04	1.10	0.03	0.17	2.66	0.008	**
Status & Wealth																
Educational capital	-0.08	0.02	0.93	-0.12	-0.03	-3.36	0.001	***	-0.08	0.02	0.92	-0.13	-0.04	-3.58	0.000	***
Market integration	-0.05	0.02	0.95	-0.09	-0.02	-3.01	0.003	**	-0.05	0.02	0.95	-0.09	-0.02	-2.96	0.003	**
Non-farming wealth	0.04	0.02	1.04	0.01	0.07	2.38	0.018	*	0.01	0.02	1.01	-0.02	0.05	0.74	0.462	
Farming wealth	0.04	0.02	1.04	0.01	0.07	2.36	0.018	*	0.03	0.02	1.03	0.00	0.06	2.14	0.033	*
Educational capital*Non-farming wealth									-0.04	0.01	0.96	-0.07	-0.02	-3.05	0.002	**
Group level predictors																
Mean Educational capital																
Mean Market integration																
Mean Non-farming wealth																
Mean Farming wealth																
Variance components																
Between-community intercept variance	0.008								0.008							
Between-individual intercept variance	0.001								0.000							
Educational capital slope variance																
Market integration slope variance																
Non-farming wealth slope variance																
Farming wealth slope variance																
Educational capital*Non-farming wealth slope variance																
Model fit and summary																
Sample size	1972								1972							
Log likelihood	-3310.70								-3306.20							
DIC	6621.38								6612.35							
Δ DIC									-9.03							

p < 0.05*, p < 0.005**, p < 0.001***

Table S4. Breakdown of the final multilevel Poisson regression model predicting individual fertility (n = 1,972) across the 22 communities, using grand-mean centered variables (A) Fixed effects only, (B) Fixed effects including the fixed interaction between non-farming wealth and educational capital.

(C) Varying slopes

Predictor	β	s.e.	exp(β)	95% CI (β)		z-value	Pr(<|z|)	
Intercept	0.94	0.03	2.57	0.88	1.00	30.57	0.000	***
Controls								
Age	0.04	0.00	1.04	0.03	0.04	24.25	0.000	***
Age^2	0.00	0.00	1.00	0.00	0.00	-18.79	0.000	***
Experienced under 5 mortality (ref = no)	0.31	0.06	1.36	0.19	0.43	5.08	0.000	***
Farmer (ref = no)	0.10	0.04	1.10	0.02	0.17	2.64	0.008	**
Status & Wealth								
Educational capital	-0.09	0.03	0.91	-0.15	-0.04	-3.24	0.001	**
Market integration	-0.05	0.02	0.95	-0.09	-0.02	-2.98	0.003	**
Non-farming wealth	0.01	0.02	1.01	-0.04	0.05	0.27	0.784	
Farming wealth	0.05	0.02	1.05	0.01	0.08	2.69	0.007	**
Educational capital*Non-farming wealth	-0.05	0.02	0.95	-0.08	-0.02	-2.86	0.004	**
Group level predictors								
Mean Educational capital								
Mean Market integration								
Mean Non-farming wealth								
Mean Farming wealth								
Variance components								
Between-community intercept variance	0.000							
Between-individual intercept variance	0.000							
Educational capital slope variance	0.005							
Market integration slope variance	0.000							
Non-farming wealth slope variance	0.003							
Farming wealth slope variance	0.000							
Educational capital*Non-farming wealth slope variance	0.001							
Model fit and summary								
Sample size	1972							
Log likelihood	-3309.00							
DIC	6617.90							
Δ DIC	5.55							

(D) Community means without varying slopes

Predictor	β	s.e.	exp(β)	95% CI (β)		z-value	Pr(<|z|)	
Intercept	0.93	0.04	2.54	0.86	1.00	26.44	0.000	***
Controls								
Age	0.04	0.00	1.04	0.04	0.04	24.59	0.000	***
Age^2	0.00	0.00	1.00	0.00	0.00	-18.75	0.000	***
Experienced under 5 mortality (ref = no)	0.31	0.06	1.37	0.20	0.43	5.19	0.000	***
Farmer (ref = no)	0.09	0.04	1.10	0.02	0.17	2.53	0.011	*
Status & Wealth								
Educational capital	-0.08	0.02	0.93	-0.12	-0.03	-3.38	0.001	***
Market integration	-0.05	0.02	0.95	-0.09	-0.02	-2.82	0.005	**
Non-farming wealth	0.02	0.02	1.02	-0.02	0.05	0.85	0.395	
Farming wealth	0.04	0.02	1.04	0.01	0.07	2.29	0.022	*
Educational capital*Non-farming wealth	-0.04	0.01	0.96	-0.07	-0.02	-3.01	0.003	**
Group level predictors								
Mean Educational capital	-0.12	0.05	0.89	-0.21	-0.03	-2.51	0.012	*
Mean Market integration	0.05	0.04	1.06	-0.03	0.14	1.33	0.183	
Mean Non-farming wealth	-0.02	0.02	0.98	-0.06	0.03	-0.79	0.427	
Mean Farming wealth	-0.06	0.03	0.94	-0.12	0.00	-1.96	0.050	.
Variance components								
Between-community intercept variance	0.004							
Between-individual intercept variance	0.000							
Educational capital slope variance								
Market integration slope variance								
Non-farming wealth slope variance								
Farming wealth slope variance								
Educational capital*Non-farming wealth slope variance								
Model fit and summary								
Sample size	1972							
Log likelihood	-3302.30							
DIC	6604.7							
Δ DIC	-13.20							

(E) Community means with varying slopes

Predictor	β	s.e.	exp(β)	95% CI (β)		z-value	Pr(<|z|)	
Intercept	0.94	0.03	2.57	0.88	1.01	27.55	0.000	***
Controls								
Age	0.04	0.00	1.04	0.04	0.04	24.59	0.000	***
Age^2	0.00	0.00	1.00	0.00	0.00	-18.72	0.000	***
Experienced under 5 mortality (ref = no)	0.31	0.06	1.36	0.19	0.42	5.01	0.000	***
Farmer (ref = no)	0.08	0.04	1.09	0.01	0.16	2.24	0.025	*
Status & Wealth								
Educational capital	-0.07	0.02	0.93	-0.12	-0.02	-2.97	0.003	**
Market integration	-0.05	0.02	0.95	-0.09	-0.01	-2.59	0.009	**
Non-farming wealth	0.01	0.03	1.01	-0.04	0.06	0.56	0.578	
Farming wealth	0.04	0.02	1.04	0.01	0.07	2.50	0.013	*
Educational capital*Non-farming wealth	-0.05	0.02	0.95	-0.08	-0.01	-2.76	0.006	**
Group level predictors								
Mean Educational capital	-0.13	0.05	0.88	-0.22	-0.04	-2.74	0.006	**
Mean Market integration	0.08	0.04	1.08	0.00	0.16	1.87	0.062	.
Mean Non-farming wealth	-0.02	0.02	0.98	-0.06	0.02	-0.92	0.356	
Mean Farming wealth	-0.04	0.03	0.97	-0.10	0.03	-1.10	0.271	
Variance components								
Between-community intercept variance	0.003							
Between-individual intercept variance	0.000							
Educational capital slope variance	0.001							
Market integration slope variance	0.001							
Non-farming wealth slope variance	0.005							
Farming wealth slope variance	0.000							
Educational capital*Non-farming wealth slope variance	0.001							
Model fit and summary								
Sample size	1972							
Log likelihood	-3298.00							
DIC	6595.97							
Δ DIC	-8.73							

$p < 0.05$*, $p < 0.005$**, $p < 0.001$***

Table S4 Continued. Breakdown of the final multilevel Poisson regression model predicting individual fertility (n = 1,972) across the 22 communities, using grand-mean centered variables (C) Varying slopes without community means, (D) Community means without varying the slopes, (E) Community means and varying slopes.

	Educational capital				Market integration				Non-farming wealth				Farming wealth				Educational capital*Non-farming wealth			
**Community ID**	**β**	**95% CI (β)**		**exp(β)**	**β**	**95% CI (β)**		**exp(β)**	**β**	**95% CI (β)**		**exp(β)**	**β**	**95% CI (β)**		**exp(β)**	**β**	**95% CI (β)**		**exp(β)**
1	-0.09	-0.20	0.02	0.91	-0.04	-0.07	-0.02	0.96	0.02	-0.05	0.08	1.02	0.05	0.03	0.07	1.05	-0.04	-0.10	0.01	0.96
2	-0.23	-0.32	-0.14	0.80	-0.02	-0.05	0.00	0.98	0.04	-0.02	0.11	1.05	0.07	0.06	0.09	1.08	0.03	-0.02	0.08	1.03
3	-0.05	-0.15	0.05	0.95	-0.04	-0.07	-0.02	0.96	-0.01	-0.08	0.06	0.99	0.04	0.03	0.06	1.05	-0.07	-0.12	-0.02	0.93
4	-0.01	-0.10	0.08	0.99	-0.08	-0.10	-0.06	0.92	0.10	0.03	0.17	1.11	0.03	0.02	0.05	1.04	-0.06	-0.11	-0.01	0.94
5	-0.06	-0.18	0.07	0.94	-0.05	-0.08	-0.02	0.96	0.00	-0.07	0.08	1.00	0.05	0.02	0.07	1.05	-0.06	-0.13	0.00	0.94
6	0.01	-0.09	0.11	1.01	-0.06	-0.08	-0.03	0.94	0.00	-0.07	0.07	1.00	0.03	0.02	0.05	1.04	-0.10	-0.15	-0.04	0.91
7	-0.03	-0.17	0.12	0.98	-0.05	-0.09	-0.01	0.95	0.00	-0.09	0.09	1.00	0.04	0.01	0.07	1.04	-0.08	-0.15	-0.01	0.92
8	-0.06	-0.15	0.02	0.94	-0.05	-0.08	-0.03	0.95	0.04	-0.03	0.11	1.04	0.05	0.03	0.06	1.05	-0.05	-0.09	-0.01	0.95
9	-0.14	-0.29	0.01	0.87	-0.03	-0.07	0.00	0.97	0.02	-0.08	0.12	1.02	0.06	0.03	0.09	1.06	-0.02	-0.10	0.06	0.98
10	-0.06	-0.16	0.04	0.94	-0.06	-0.09	-0.03	0.94	0.06	-0.02	0.14	1.06	0.04	0.03	0.06	1.05	-0.05	-0.10	0.01	0.96
11	-0.15	-0.27	-0.04	0.86	-0.03	-0.06	0.00	0.97	0.01	-0.07	0.09	1.01	0.06	0.04	0.08	1.06	-0.01	-0.08	0.05	0.99
12	-0.11	-0.19	-0.03	0.90	-0.04	-0.06	-0.02	0.96	0.01	-0.05	0.07	1.01	0.05	0.04	0.07	1.06	-0.04	-0.08	0.01	0.97
13	0.00	-0.09	0.10	1.00	-0.08	-0.10	-0.05	0.92	0.08	0.02	0.15	1.09	0.03	0.02	0.05	1.03	-0.07	-0.12	-0.02	0.93
14	0.12	0.01	0.24	1.13	-0.09	-0.12	-0.05	0.92	0.03	-0.05	0.11	1.03	0.01	-0.01	0.03	1.01	-0.14	-0.20	-0.09	0.87
15	-0.16	-0.30	-0.02	0.85	-0.03	-0.07	0.00	0.97	0.03	-0.06	0.13	1.03	0.06	0.04	0.09	1.06	-0.01	-0.08	0.07	0.99
16	-0.08	-0.23	0.07	0.92	-0.06	-0.09	-0.02	0.95	0.06	-0.03	0.15	1.06	0.05	0.02	0.07	1.05	-0.03	-0.11	0.04	0.97
17	-0.17	-0.26	-0.07	0.85	-0.03	-0.06	-0.01	0.97	0.04	-0.04	0.11	1.04	0.06	0.05	0.08	1.07	0.00	-0.05	0.05	1.00
18	-0.12	-0.23	-0.01	0.89	-0.05	-0.08	-0.02	0.95	0.07	-0.01	0.16	1.08	0.05	0.04	0.07	1.06	-0.01	-0.07	0.05	0.99
19	-0.06	-0.17	0.06	0.94	-0.05	-0.08	-0.02	0.95	0.03	-0.05	0.10	1.03	0.05	0.03	0.06	1.05	-0.06	-0.12	0.01	0.95
20	-0.12	-0.18	-0.06	0.89	-0.02	-0.04	0.00	0.98	-0.05	-0.10	0.00	0.95	0.06	0.05	0.07	1.06	-0.05	-0.08	-0.02	0.95
21	-0.10	-0.26	0.07	0.91	-0.05	-0.09	-0.01	0.95	0.04	-0.05	0.13	1.04	0.05	0.02	0.08	1.05	-0.03	-0.12	0.05	0.97
22	-0.04	-0.15	0.07	0.96	-0.06	-0.10	-0.03	0.94	0.06	-0.01	0.13	1.06	0.04	0.02	0.06	1.04	-0.05	-0.11	0.00	0.95
Fixed Effect	-0.08	-0.14	-0.02	0.93	-0.05	-0.08	-0.01	0.95	0.03	-0.01	0.08	1.03	0.05	0.02	0.08	1.05	-0.04	-0.08	-0.01	0.96

Table S5. Varying coefficients, 95% CIs and exponentiated coefficients for each of the four wealth and status measures, and the interaction between educational capital and non-farming wealth, in each community

Full model with childless women removed - post-reproductive women only

Predictor	β	s.e.	exp(β)	95% CI (β)		z-value	Pr(<|z|)	
Intercept	1.18	0.06	3.25	1.06	1.30	19.45	0.000	***
Controls								
Relative age	0.00	0.01	1.00	-0.01	0.01	-0.18	0.854	
Relative age^2	0.00	0.00	1.00	0.00	0.00	0.94	0.350	
Experienced under 5 mortality (ref = no)	0.25	0.07	1.28	0.12	0.38	3.76	0.000	***
Farmer (ref = no)	0.09	0.05	1.09	-0.01	0.18	1.76	0.078	.
Status & Wealth								
Relative Educational capital	-0.08	0.03	0.92	-0.14	-0.03	-2.94	0.003	**
Relative Market integration	-0.02	0.02	0.98	-0.06	0.02	-0.81	0.416	
Relative Non-farming wealth	-0.03	0.02	0.97	-0.08	0.02	-1.32	0.187	
Relative Farming wealth	0.05	0.02	1.05	0.01	0.09	2.67	0.008	**
Relative Educational capital*RelativeNon-farming wealth	-0.04	0.02	0.96	-0.08	-0.01	-2.31	0.021	*
Group level predictors								
Mean Educational capital	-0.08	0.05	0.92	-0.18	0.02	-1.56	0.118	
Mean Market integration	0.01	0.04	1.01	-0.08	0.09	0.13	0.897	
Mean Non-farming wealth	-0.02	0.02	0.98	-0.06	0.03	-0.75	0.452	
Mean Farming wealth	-0.04	0.03	0.96	-0.11	0.02	-1.31	0.191	
Variance components								
Between-community intercept variance	0.004							
Between-individual intercept variance	0.000							
Model fit and summary								
Sample size	864							
Log likelihood	-1710.90							
DIC	3421.84							

$p < 0.05$*, $p < 0.005$**, $p < 0.001$***

Table S6. Full model run on all post-reproductive women, where all childless women have been removed from the analysis (*n* = 864).

Full model with childless women removed - all parous women included

Predictor	β	s.e.	exp(β)	95% CI (β)		z-value	Pr(<|z|)	
Intercept	1.05	0.03	2.85	0.98	1.11	32.83	0.000	***
Controls								
Relative age	0.02	0.00	1.02	0.02	0.02	11.47	0.000	***
Relative age^2	0.00	0.00	1.00	0.00	0.00	-6.25	0.000	***
Experienced under 5 mortality (ref = no)	0.24	0.06	1.27	0.12	0.35	3.91	0.000	***
Farmer (ref = no)	0.07	0.04	1.08	0.00	0.15	1.92	0.054	.
Status & Wealth								
Relative Educational capital	-0.07	0.02	0.93	-0.12	-0.03	-3.30	0.001	***
Relative Market integration	-0.01	0.02	0.99	-0.05	0.02	-0.83	0.409	
Relative Non-farming wealth	-0.02	0.02	0.98	-0.06	0.02	-1.10	0.272	
Relative Farming wealth	0.05	0.02	1.05	0.02	0.08	3.08	0.002	**
Relative Educational capital*RelativeNon-farming wealth	-0.04	0.01	0.96	-0.07	-0.01	-2.92	0.004	**
Group level predictors								
Mean Educational capital	-0.12	0.04	0.89	-0.19	-0.05	-3.37	0.001	***
Mean Market integration	0.03	0.03	1.04	-0.02	0.09	1.22	0.221	
Mean Non-farming wealth	-0.02	0.02	0.98	-0.05	0.01	-1.28	0.199	
Mean Farming wealth	-0.04	0.02	0.96	-0.09	0.01	-1.60	0.109	
Variance components								
Between-community intercept variance	0.001							
Between-individual intercept variance	0.000							
Model fit and summary								
Sample size	1,538							
Log likelihood	-2759.40							
DIC	5518.74							

$p < 0.05$*, $p < 0.005$**, $p < 0.001$***

Table S7. Full model run on all parous women in the sample (*n* = 1,538).

Supplementary References

1. Kolenikov, S. & Angeles, G. 2009 Socioeconomic Status Measurement with Discrete Proxy Variables: is Principal Component Analysis a Reliable Answer? *Rev. Income Wealth* **55**, 128–165. (doi:10.1111/j.1475-4991.2008.00309.x)

2. Costello, A. B. & Osborne, J. W. 2005 Best Practices in Exploratory Factor Analysis: Four Recommendations for Getting the Most From Your Analysis. *Pract. Assessmenr, Res. Eval.* **10**, 1–9.

3. Braveman, P. A., Cubbin, C., Egerter, S., Chideya, S., Marchi, K. S., Metzler, M. & Posner, S. 2005 Socioeconomic status in health research: one size does not fit all. *JAMA* **294**, 2879–88. (doi:10.1001/jama.294.22.2879)

4. Mulder, M. B. & Beheim, B. a 2011 Understanding the nature of wealth and its effects on human fitness. *Philos. Trans. R. Soc. Lond. B. Biol. Sci.* **366**, 344–56. (doi:10.1098/rstb.2010.0231)

5. Borgerhoff Mulder, M. et al. 2009 Intergenerational wealth transmission and the dynamics of inequality in small-scale societies. *Science (80-.).* **326**, 682–688. (doi:326/5953/682 [pii] 10.1126/science.1178336)

6. Chung, Y., Rabe-hesketh, S., Gelman, A., Dorie, V. & Liu, J. 2011 Avoiding Boundary Estimates in Linear Mixed Models Through Weakly Informative Priors. *Berkeley Prepr.*, 1–30.

7. Chung, Y., Rabe-hesketh, S., Gelman, A., Liu, J. & Dorie, V. 2012 A non-degenerate estimator for hierarchical variance parameters via penalized likelihood estimation. *Psychometrica*, 1–34.

8. R Core Team 2014 R: A language and environment for statistical computing. R Foundation for Statistical Computing, Vienna, Austria. URL http://www.R-project.org/.

9. Bates, D., Maechler, M. & Bolker, B. 2012 lme4: Linear mixed-effects models using S4 classes.

10. Gelman, A., Su, Y.-S., Yajima, M., Hill, J., Pittau, M. G., Kerman, J., Zheng, T. & Dorie, V. 2013 arm: Data Analysis Using Regression and Multilevel/Hierarchical Models.

11. Dorie, V. 2013 blme: Bayesian Linear Mixed-Effects Models.

12. R. H. Baayen 2011 languageR: Data sets and functions with ‘Analyzing Linguistic Data: A practical introduction to statistics’.

13. Handcock, M. S. 2013 reldist: Relative Distribution Methods.

14. Kreft, I. G. G., de Leeuw, J. & Aiken, L. S. 1995 The Effect of Different Forms of Centering in Hierarchical Linear Models. *Multivariate Behav. Res.* **30**, 1–21. (doi:10.1207/s15327906mbr3001_1)
